# Pathology, Practical Medicine, and Therapeutics

**Published:** 1837-07

**Authors:** 


### PATHOLOGY, PRACTICAL MEDICINE, AND THERAPEUTICS.

### *On the External Application of Opium in Bronchitis and Croup.* By Dr. Bow, of Alnwick.

DR. Bow has been in the habit of employing opium externally, on an extensive scale, for some years, in the treatment of some internal diseases; and its benefits in the bronchitis and croup in children are, according to his report, really wonderful. "These diseases," he says, "are suddenly and effectually subdued by this simple means. In a few minutes after the first application, there will in most cases be a decided amendment. In some cases, with well-marked symptoms, one application has been sufficient to effect a cure." He adds, that he has had six or seven years' experience of this plan of treatment, and has not lost a single case from bronchitis. The opium is formed into a liniment as follows: ℞. Opii, ℥j.; Saponis, ℥ss.; Liniment. Camph. comp. ℥viij. M. Digere per aliquot dies; and rubbed upon the chest. The process is described by Dr. B. as follows: "I order the child to be laid across the nurse's knee, with the head depending, so that the ammoniacal vapour from the liniment may not affect the nostrils and eyes. I then pour the liniment on the breast in such quantity as to require a brisk motion of the nurse's hand, to prevent it from running down the sides. This she applies to the breast, neck, and bowels; and, as it dries, I continue to renew it, until from two to four drachms, or thereabouts, have been used. An hour afterwards, if there be no amendment, I repeat it, applying the liniment also to the back and limbs."

It must be confessed that the success here announced is not a little startling to us as practitioners of some experience, and, like our brethren, too often witnesses of the dangerous consequences of pulmonary inflammation in infants. As Dr. Bow, however, details what he has seen, we request our readers' attention to the practice, which we ourselves purpose putting to the test, in the first case of bronchitis that we meet with. We own we shall not be disappointed if we meet with a much less degree of success than that recorded by Dr. Bow.

It is but justice to Dr. Bow, as a rational practitioner, to state that his practice is founded on peculiar views of inflammation, and that he believes infantile bronchitis, in particular, would be cured, "if left to nature and good nursing," in forty-nine cases out of fifty; while, when treated as an inflammatory disease, by the lancet, "two out of three perish."

*Lancet. March* 18, 1837.

### *On Castor-Oil Frictions in Gout.* By W. E. POPE, Esq.

MR. POPE reports the effect of this remedy on the authority of a friend, an officer resident in India. This gentleman says that it has been tried in the East with the greatest success, and that he himself had cured two or three cases of gout by it, after other remedies had failed. The mode of applying it is to rub in the oil at bedtime into the affected limbs, and then wrap them up in warm flannels. The remedy is said to act like a charm, seeming to exert a specific influence in curing the disease. We wish we could believe in the efficacy of this simple treatment.

*Lancet. April* 8, 1837.

---

### *On Nux Vomica in Affections of the Digestive Organs.* By Thomas Mellor, Esq., Surgeon, Manchester.

Mr. Mellor informs us that he has been in the habit of employing nux vomica in disorders of the digestive functions for several years, and with the greatest benefit. The cases in which it has been most beneficial are those of pyrosis from functional disorder of the stomach, in which Mr. M. considers it as "almost a specific; so immediate, and in most instances so prominent, has been the relief obtained." In pyrosis from organic causes it is very useful. In almost all cases of dyspepsia, also, not complicated with inflammation of the mucous membrane, but where the stomach is obviously debilitated, as indicated by pain and distention after eating, flatulence, &c., Mr. M. considers the remedy as advantageous. He exhibits the nux vomica in the form of powder, in doses of three, four, or five grains, suspended in cinnamon water, with mucilage and some tincture of cardamoms, thrice daily. He has never found any ill effects from it. *Med. Gazette. March* 4, 1837.

---

### *An Account of Tubercles in the Air-Cells of a Bird, and some Observations on Tubercles in general.* By R. Harrison, M.D., &c.—*Dublin.*

This observation is interesting, and is as greatly in favour of Dr. Carswell's views as it is strongly against the views of Mr. Carmichael, respecting the origin of tubercles in general. It is justly observed by Dr. Harrison, that the occurrence of the tubercles in the more open structure of the great air-cells of the bird (in the present case a male gannet,) enables us to trace many of the characters of tubercles more distinctly than in the more complex tissues in which it is usually found in the higher animals.

This bird lived a month in captivity before he died, and sunk obviously from general cachexia and debility. No peculiar affection of the breath was observed.* The following is Dr. H.'s account of the dissection.

"On raising the sternum I was at once struck with the number of yellow, white, or greyish tubercles with which the interior of the great air-cell on the left side was studded. The membrane, as it lines the ribs, (corresponding to pleura,) and thence extends as an imperfect septum between the thoracic and abdominal viscera, was thickly set with them. In size, form, and consistence, they presented every variety; some were small and circular as a pin's head, others as large as a sixpence, some were of an irregular or diffused form, but the circular shape prevailed; many were very firm, and almost dry, others more soft and pulpy to the feel, and several were semi-fluid about their circumference: in many the centre was depressed, and as it were shrivelled in the form of a dry circular nucleus, while in a few the same part was very prominent and conical, but dry and apparently inorganic or horny. Those of the smallest size were of softest consistence, but even these consisted of the same white or yellowish matter. There were only two spots that presented any resemblance to the grey semi-transparent tubercle so common on the human lung. Some of this numerous crop of tubercles adhered very closely to the membrane

* Dr. H. expresses his belief that birds have never been observed to cough or sneeze, and that "they are physically incompetent to make either of these convulsive expiratory efforts." We can only say that a tame cocatoo, now perched near us while we are writing, and who is very subject to coryza, appears to sneeze frequently and fully: we cannot, however, positively assert that there is no voluntary effort combined in the operation.—Ed.

which may be supposed their matrix, others less intimately, and some were so loosely connected that I lost several when passing a gentle stream of water over the surface for the purpose of cleaning the preparation. The softening and separation in all cases extended from the circumference inwards; the connexion appeared a mere agglutition, which a little maceration in water would easily dissolve; the surface of the subjacent membrane appeared free from any abrasion or abnormal appearance. The subject had been tolerably minutely injected, yet not a trace of blood-vessel could be detected either entering any of these tuberculous masses or connected to their base, and there was no vestige of inflammation, recent or remote."

A considerable portion of Dr. Harrison's paper is taken up in endeavouring to prove, in opposition to Dr. Carswell's opinion, that the *serous* membranes are more frequently the seat of tubercles than the *mucous* are; and, in order to reconcile the fact of their occurrence in the pulmonary cells, he attempts to show that these are in reality lined by a serous, and not by a mucous membrane, as is commonly supposed. *Dublin Journal. May,* 1837.

---

*On the Tonic Treatment of Erysipelas.* By HENRY BULLOCK, Esq.

THE author adduces a few cases from the wards of St. Thomas's Hospital, to prove the contagious nature of erysipelas, and seems to consider that the establishment of this quality of the disease almost necessarily establishes the impropriety of antiphlogistic treatment, and the propriety of the tonic treatment by wine, &c. Our readers will not receive this as fair reasoning, neither will such of them as have experience doubt (as Mr. Bullock does,) that the same disease may require a different treatment in different situations and circumstances. Mr. Bullock informs us, that, from January to June inclusive, last year, thirty-two cases of erysipelas occurred in St. Thomas's Hospital, and were treated by wine, either alone or combined with tonics, from the first appearance of the disease, and all except two recovered. This certainly, as far as it goes, is evidence in favour of the mode of treatment pursued by Dr. Williams in that hospital.

*Med. Gazette. March* 4, 1837.

---

*On the "Bruit du Diable."* By T. O. WARD, M.D., Physician to the Birmingham Dispensary.

THE stethoscopic sound first described by M. Bouillaud under this absurd name, which we hope will not be naturalized among us, is chiefly heard in chlorotic and nervous subjects, over the course of the left carotid and subclavian, above the inner end of the clavicle. M. Bouillaud considers it to be intermediate between the bellows murmur and the musical sound of the arteries described by Laennec, and attributes it partly to a watery state of the blood, as it ceases to be audible on the return of health; partly to the rapid circulation so near the heart; and partly to the vicinity of the larynx, which acts as a sort of sounding board. As its name implies, the sound resembles that of a humming-top. A knowledge of it is rather of negative than positive value, as it might mislead the young observer to suspect organic disease when none exists. M. Bouillaud says he has observed above a hundred cases of it in the three years during which it has engaged his attention. Dr. Ward has met with five cases of it; and the object of the present paper is chiefly to assign a site for the sound different from that alleged by M. Bouillaud. Dr. Ward conceives the sound to be produced exclusively *by the veins;* but he fully agrees with M. Bouillaud in referring it to a too fluid state of the blood, with increased rapidity of the circulation. Dr. W. adduces the following arguments in support of his opinion:

" First, the sound has been heard over the course of the external jugular vein, as well as over the carotid arteries. Secondly, it is solely in this latter situation, and not always there, that the sound is so modified by the proximity of the carotid as to appear to be augmented by the ventricular systole. Thirdly, it is arrested by pressure on the external jugular, when heard along the course of that vessel; and, as is asserted by M. B., by a pressure over the carotid quite insufficient to stop the pulsation of the artery, but which would certainly obstruct the flow of blood through the internal jugular. Fourthly, it is increased by pressure, and by everting the head with the chin raised; because, under these circumstances, the caliber of the vein is diminished, and consequently the velocity of the current is increased. Sixthly, for the contrary reason, the sound is stopped by turning the head so as to shorten the vein and retard the course of its contents towards the heart. The greater distance the blood has to traverse in passing from the left side of the neck into the vena cava, will also explain the greater frequency of the sound on that side. Seventhly, the sound is arrested by pushing aside the larynx, because this can be accomplished only while the muscles of the fore part of the neck are relaxed, at which time the jugular vein is relaxed also; besides that, the larynx and trachea sometimes prolong, by their resonance, the pulsations of the carotid and the respiratory murmur, so as to simulate the bruit du diable when none exists. Eighthly, a violent or a prolonged effort produces regurgitation in the veins, and thus puts a stop to the flow and sound of their contents. When the sound intermits, I have been able to account for it by the pressure of the stethoscope, or by the position of the patient's head. Tenthly, the reason that the purring tremor, when felt, is slighter in these cases than when a contraction of the cardiac orifices exists, arises from the difference in the strength and tension of the vessels, by whose vibrations the phenomenon is produced."

The profession is much indebted to Dr. Ward for calling attention to this sound, for which we hope he will find an English name. We have ourselves always regarded it as a mere variety of the Laennecian sounds, and as having its origin in the arterial current: we do not feel quite convinced of our error by the ingenious reasoning of Dr. Ward. *Med. Gazette. April* 1, 1837.

---

*Taste of Quina* Mr. Sherwin, of Hull, says that a bit of apple, chewed for a moment, will in an instant efface the bitter taste of sulphate of quinine.

*Med. Gazette. April* 1, 1837.

---

*Remarks on the Treatment of Hydrocephalus Acutus.* By Dr. Mayo.

This paper was read at one of the evening meetings of the College of Physicians. It contains nothing new; but it is a very valuable and interesting digest of the principles and practice of Whytt, Carmichael Smyth, Clarke, Dobson, Gölis, and Abercrombie, in hydrocephalus, interspersed with sensible and judicious critical remarks of the author. It passes in review, with much impartiality, the actual and relative value of bleeding, purging, mercury, and diuretics; and indicates the cases and stages in which they are respectively most applicable and useful. The essay does not admit of extract, but we recommend it to the attention of the young practitioner. The following observation ought to be present to the mind of every one called on to treat this most fatal disease: "Compared with the whole duration of the disease, the curable portion of it is very short, the mortuary portion very long. Hence it happens that the time for action has often elapsed before we are summoned; while, again, if we bring with us indeterminate opinions as to its treatment, the disease will have gained a victory before we have resolved upon the weapons with which it is to be encountered."

*Med. Gazette. April* 8, 1837.

---

*On the Influence of Gravity on the Circulation of the Blood.*
By F. R. Mosely, Esq., Surgeon.

The object of this short paper is to call the attention of practitioners to the actual and probable effect of the posture of the body in the production and relief of diseases, by influencing the venous circulation, particularly in the head. The author says that it may be regarded as generally true, that, whenever the venous circulation in any part of the body is favoured by the gravity of the blood under ordinary circumstances, congestion will be apt to take place from any change of the accustomed position. Thus, in the horizontal position, the veins of the neck and head, which are nearly passive tubes, become easily distended; and in this manner, Mr. M. contends, cerebral and nervous diseases are often induced, aggravated, or perpetuated. He says he has observed severe cephalic disorders emanating from this cause among the poor, from sleeping without a pillow; and in elderly people long confined to bed, from whatever cause, he thinks he has not unfrequently observed obstinate nervous symptoms, which he thinks may probably originate in the altered state of the cephalic circulation, from the prolonged horizontal posture. We think these observations deserve attention. The influence of even a slight change of position in altering the circulation within the head must have been observed by every one in his own person: merely bringing the lower extremities from the dependent to the horizontal position, without changing the elevation of the trunk of the body,—as in turning from the sitting to the semi-recumbent posture on the sofa, by lifting up the legs,—greatly disposes to sleep, no doubt from this cause; and every practitioner must have found happy effects, in certain nervous diseases, from inducing the patient to sit up. Mr. Mosely says, that great benefit will be found in the treatment of fever from an apparatus placed under the pillow, to regulate the elevation of the head at pleasure.

The author also speculates on the effect of gravity in producing and aggravating pulmonary and other visceral diseases; and we have no doubt that his conjectures are just to a certain extent. He thinks that the comparative frequency of pneumonia on the left and on the right side, and also of congestion of the liver and spleen, may be possibly influenced by the habitual position of the patients in bed. He says, that "the mere confinement to the bed appears often to bring on cough and pectoral symptoms among old and infirm patients," not to be relieved so long as they retain the horizontal position. He might have added, that Laennec, in his account of what he terms *Pneumonie des agonisans*, says, that the frequent supervention of inflammation of the lung in such cases arises from the congestion produced by the imperfect circulation in the lungs; and this is strongly dwelt on as a cause, by MM. Hourmann and Dechambre, as a common cause of the disease in old persons, not moribund.

*Med. Gazette. April* 15, 1837.

---

*Clinical Report of the Cases treated in the Fever Ward, No.* 9, *of the Royal Infirmary, Edinburgh, during the Year* 1836-7. By DAVID CRAIGIE, M.D. F.R.S.E.

THIS is a very excellent report, drawn up with great accuracy, and containing much important matter, statistical, pathological, and practical: it includes all the cases treated from the 28th June, 1836, to the 12th February, 1837. We can only find room for a few extracts from the third part, entitled *General Summary.*

The whole number of cases treated amounted to 181; but from these, twelve cases are deducted, as not being cases of fever. In like manner, the actual deaths were thirty-one; but from this number eight are deducted, for the same reason. Allowing for these deductions, the number of fever cases, properly so called, are 169; of this number, twenty-three proved fatal,—i. e. 1 in 7⅓, or 135 per 1000.

Among the whole 169, in only fifty-four cases could the disease be with probability traced to contagion; in one, it was traced to contagion and a blow on the head; in two, to contagion doubtfully; in forty-seven it was imputed to cold, or to cold and moisture; and in two it was traced to drinking.

The day of the disease on which the patients applied for admittance was very various; in a few cases early, in general after several days' illness; the great majority were admitted from the fourth to the eighth day; the largest number on this last.

The greatest number of cases was admitted between the ages of ten and twenty, and twenty and thirty; those between thirty and forty amounted to not quite half the latter; and the numbers admitted below ten and above forty were very nearly the same.

The following interesting results and observations conclude the Report:

"Upon examining the cases, with the view of ascertaining to what extent local affections of particular regions and organs prevailed, and classifying the cases treated according to the affection of particular organs, we find that, in a very small number of instances (2) was the febrile disorder free from symptoms of local complaint, and that in many cases more than one organ was affected. To give some idea of the complication of the febrile process, I have classified the whole of the cases treated and presenting symptoms of fever, in the following manner:

| | Cases. |
|---|---|
| Symptoms of affection of the head alone occurred in . . . . | 47 |
| Symptoms of affection of the thoracic viscera, chiefly the lungs alone, occurred in . . . . . . . | 5 |
| Symptoms of affection of the epigastric organs alone occurred in . | 7 |
| Symptoms of affection of the throat alone occurred in . . . | 1 |
| Symptoms of affection of the abdominal organs without diarrhœa occurred in | 1 |
| Symptoms of affection of the abdominal organs with diarrhœa occurred in | 2 |
| Symptoms of affection of the head and chest, occurred in . . | 23 |
| Symptoms of affection of the head and epigastric region in . . | 59 |
| Symptoms of affection of the chest and epigastric in . . . | 1 |
| Symptoms of the head, chest, and epigastric region in . . | 6 |
| Typhomania and gangrene of the toes took place in . . . | 1 |
| Gangrene of the lungs took place in . . . . . | 1 |
| Symptoms of head disorder with double parotids, took place in . | 3 |
| Symptoms of head disorder with single parotids, in . . . | 3 |
| Symptoms of affection of the head, throat, chest, and belly . . | 1 |
| Symptoms of affection of the head, throat, and chest, in . . | 3 |
| Symptoms of affection of the head, throat, and epigastrium, in . . | 2 |
| Symptoms of affection of the head with diarrhœa . . . | 1 |
| Fever without local affection . . . . . . | 2 |
| Fever with anorexia and articular pains . . . . . | 1 |

"From these numerical statements several important conclusions, illustrating the predominant characters of fever in this country, may be deduced.

"1. The first proposition to which these statements lead, is, that by far the most frequent form which fever assumes in this country consists in more or less pain of the head, generally referred to the frontal region, with suffusion of the eyes, which are turbid and watery, and more or less intolerant of light,—accompanied, further, with tenderness or pain in the epigastric or umbilical region, or both, some tension in that region, sometimes commencing with the frontal headach, sometimes following it, but always felt more or less intensely in the course of the disease. This combination of symptoms took place in more than one-third of the whole number of cases.

"2. The second proposition which results from the comparison of the numbers now given, is, that the next frequent form which fever assumes, is that in which symptoms of derangement in the cerebro-meningeal circulation take place, either alone, or in such an intense form as to obscure or mask every other local disorder. This form of the disease occurred in about two-sevenths of the whole number of cases.

"3. The complication which follows next in point of frequency is that in which the symptoms of fever are associated with those of disorder of the cerebro-meningeal circulation, and at the same time of some affection of the bronchial membrane, tubular or vesicular, or of the lungs. This complication took place in more than one-seventh of the cases. It will afterwards appear that the numbers show that this is by far the most fatal variety of complication.

"4. The class of cases next in frequency consists of those in which there were symptoms of tenderness or pain, with or without tension in the epigastric or umbilical region. This took place in one twenty-fourth part of the cases.

"5. Not very different is the proportion of cases which present at once symptoms of disorder in the head, chest, and epigastric region, being about one twenty-eighth part of the whole amount.

"*Lastly*, it appears that the number of intestinal affections is exceedingly small, since symptoms of disorder in the intestinal mucous membrane took place in two cases only, and of uneasiness of the abdomen with *stridor dentium*, probably from irritation of the intestinal membrane, appeared in one case only.

"From these numerical statements several important results, as to the occurrence of fatal cases, and the circumstances by which the fatal termination is influenced, may yet be deduced.

"In the first place, it appears that by far the largest proportion of fatal cases is found in that class which presented the twofold affection of the head and chest. Of the twenty-three cases constituting this class, there are not fewer than seven fatal cases, or one-third—and constituting one-third of the whole mortality.

"In the next place, it appears that a large proportion of fatal cases is found among those in which the symptoms of disorder of the head, chest, and epigastric region were associated. Of the six cases arranged under this head, three or one-half were fatal. This constitutes nearly one-eighth part of the whole mortality. Under the same head may be arranged the only case in which the symptoms of disorder were referred at once to the head, throat, chest, and belly, and which also proved fatal.

"It is remarkable, on the other hand, that, among the cases in which the symptoms of local disorder were confined to the head alone, the proportion of fatal cases is smaller than in either of the two other classes. Among the forty-seven cases referred to this head are found five fatal cases, or about one-ninth, and forming of the whole mortality less than one-fourth.

"Among the large class of cases, again, amounting to fifty-nine, in which the leading symptoms were referred to the head and epigastric region, the fatal cases were not more than seven or rather fewer than one-eighth, but forming one-third nearly of the whole mortality.

"The only class of cases in which no fatal case is found, is that in which the symptoms were confined to uneasiness, tenderness or pain in the epigastric region with or without distension."

*Edinburgh Med. and Surg. Journal. April*, 1837.

---

### *Remarks on the Physiological and Therapeutical Effects of Colchicum.* By ROBERT LEWINS, M.D.

PERHAPS the more scientific tendency of medical investigation, during the last thirty years, has somewhat misled the attention of practitioners from the careful study and just appreciation of the effects of individual remedies on the animal economy, in health and disease. Taking pathology for our guide, we have too often overlooked, in our practice, the value of those remedies, the precise agency of which is not explicable on certain received principles. Although the result of this has been a most beneficial simplification of our therapeutics, yet it has not seldom led to the neglect of remedies which experience had established as valuable, although their mode of action was unknown. It is for this reason that we are always pleased to see attempts to illustrate the therapeutical value of individual medicines; as, after all, pathological knowledge is barren unless it leads to surer indications of practice, and practice can never be precise or generally successful where the power of the agents it employs is undetermined. We are, accordingly, thankful to Dr. Lewins for having, in the present paper, called the attention of the profession to the examination of the powers and mode of action on the animal economy of one of our most valuable and energetic remedies. We think, however, that Dr. Lewins is mistaken when he states that "a great proportion of the medical profession in Great Britain have never even used this article in any disease, and are ignorant of its medicinal virtue, or at least underrate its efficacy." On the contrary, we think that in this part of the island no remedy is more frequently used or better appreciated by all intelligent and experienced practitioners; and we believe we could refer him to more than one authority for its use in the very cases, and on the same principles, as he has himself employed it.

The object of Dr. Lewins, on the present occasion, is to illustrate the effect of colchicum in fever; and his paper contains the detail of five cases of this disease treated with this remedy. Previously to using colchicum on the sick, Dr. L. made trials of its effect on healthy individuals; and certainly the most interesting portion of his communication is the account of these trials. In all the cases, the vinous tincture of the seeds was the form used.

*First Series of Cases. (Healthy Individuals.)* In the *first case,* 160 drops were taken by a youth, aged eighteen, in twenty-four hours; the first dose being fifty drops, and the last sixty. The result was, seven very copious stools, loss of appetite and debility for twenty-four hours.—2*d Case.* A youth of seventeen took 170 drops in nine hours, in doses of seventy, thirty, thirty, and forty drops: nausea and vomiting, and six copious stools, were the result.—3*d Case.* A youth, aged fifteen, took 130 drops in ten hours, and four doses; the first of forty, and the last three of thirty drops. Vomiting, and only one stool, were the consequence.—4*th Case.* A youth, aged twelve, took sixty drops in two doses, after an interval of eight hours. Nine copious watery stools were produced.—5*th Case.* A youth, aged seventeen, took forty drops at bedtime, thirty drops next morning, and thirty drops seven hours after; in all, 100 drops in nineteen hours. Vomiting and faintness, and five copious stools, were the result. The same boy afterwards took seventy drops at one dose, which was followed by vomiting and headach, but not by purging.—6*th Case.* A boy, aged ten, took eighty drops in twenty-four and a half hours, in four doses, of twenty, fifteen, twenty-five, and twenty drops. Great sickness and vomiting, and nine purgative motions, were the result.

In all these cases, the debility and feeling of illness soon disappeared after the action of the medicine.

*Second Series. (Fever Cases.)* All these were apparently slight, and such as we find to yield, in no long space of time, either to the remedial powers of nature or to common purgatives and refrigerants. They were all treated by colchicum, and in most of them convalescence seemed to set in shortly after the operation of the remedy as an emetic or cathartic, or both. In the *first case,* the colchicum was given in doses, first of thirty, and then of fifteen and ten drops, every three or six hours. Half an ounce was taken in one week, and no other medicine.—In the *second case,* forty drops were given at first, and thirty every three hours: 335 drops were taken in three days.—In the *third case,* the medicine was ordered in the same doses, but was obliged to be omitted, and other remedies used.—In the *fourth case,* the medicine was only continued during two days, and 100 drops were taken.—In the *fifth case,* sixty drops were first given, and then forty, and afterwards thirty every third hour. Half an ounce was taken in the course of forty-eight hours.

Dr. Lewins concludes the history of these cases with the following remarks, which we think may be applicable to certain forms of acute sporadic fever, but certainly not to fever generally. None of Dr. Lewins's cases had any character of typhus, and we believe he will find the results of his treatment in this form of fever very different from those exhibited in these here detailed.

"I shall conclude this communication by remarking, that, from the phenomena and results of the cases now adduced, I am convinced we may more certainly cut short fever, or at least break its force, by the judicious administration of colchicum, than by any other known means. I by no means, however, recommend that it should be trusted to exclusively in fever. Other medicines and means may, if deemed necessary or advisable, be employed, such as bloodletting, antimonials, the warm bath, &c. I, however, am disposed to think that these will seldom be required, if colchicum be boldly but prudently prescribed in the early stages of the disease. It is corroborative of my opinion, as to the efficacy of this potent drug, that its action on the animal economy is consistent with its therapeutical agency. The immediate effects produced by colchicum on the stomach, the liver, the skin, and on the circulation, are such as would lead us, *a priori,* to expect that relief would be given to that state of the system which the most rational theory of fever presumes to exist."

We recommend to the notice of Dr. Lewins, and to our readers generally, a most excellent work of Dr. Barlow, "On the Bath Waters, Gout, &c." published in 1822; also to extracts from it, in the article GOUT, in the "Cyclopædia of Practical Medicine," written by the same distinguished physician: in these treatises, Dr. Lewins will find the action and therapeutic value of colchicum stated in a manner that will convince him that this author, at least, is fully aware of the principles on which this potent remedy should be prescribed.

*Edinburgh Journal. April*, 1837.

---

*On the Cure of Headach by the Application of Leeches to the Schneiderian Membrane.* By JOHN WALKER, M.D.

THIS mode of local depletion in affections of the head has, we believe, become more frequent since the publication of Mr. Wardrop's treatise. It is certainly very beneficial in certain cases, and is likely to be the more accredited because it is one of nature's ordinary remedies in congestion in this region. Every practitioner must have witnessed relief from severe headach, by the spontaneous flow of a very small portion of blood from the nostrils. We had occasion to notice a very striking case of this kind during the late influenza, in the person of a medical friend. Dr. Walker says, he has "found much benefit from this mode of treatment in the painful headaches peculiar to pregnant females; in those arising from irregularity or total suppression of the catamenia in plethoric habits; and in a few depending upon biliary derangement." Five cases of headach are briefly related, in which from one to four leeches, applied to the membrane on each side of the septum of the nose, were productive of immediate and permanent relief.

*British Annals of Medicine. March* 31, 1837.

---

*Case in which seven Half-Crowns had been swallowed.* By H. WAKEFIELD, Esq.

A MAN, sentenced to three years' imprisonment, swallowed seven half-crowns, from fear of their being taken from him. They produced no bad effect, and the matter was forgotten; when, more than twenty months afterwards, he came under Mr. W.'s care, complaining of slight pain and tenderness of the abdomen, and, while taking some ordinary medicine, one day the whole seven pieces "fell clattering in one motion into the closestool pan." This case is curious, as showing how little disturbance, in some cases, extraneous bodies excite within the bowels."

*Med. Gazette. May* 20, 1837.

---

*On the Treatment of various Diseases by means of Creosote.* By SIR FRANCIS SMITH, M.D. &c., Dublin.

SIR F. SMITH has only employed creosote as an external application. He has successfully used it in Cancrum oris, and is "almost inclined to consider it a specific in Tinea capitis;" but the only details he gives are three cases of ulcer, in which its use as a lotion (undiluted,) was evidently most beneficial. The first case was one of primary foul and obstinate chancre; the second, fistula in ano; the third, obstinate ulceration of the mucous membrane of the nose: the cure in all was rapid, after recourse was had to the creosote.

[In making this extract from the pages of our very estimable contemporary, we cannot refrain from expressing our opinion that the learned editors are, in general, not sufficiently rigid castigators of the *style* of the valuable papers received into their Journal. If communications are hurriedly or slovenly written, we apprehend that an editor is not only justified, but called upon, by his office, to amend and polish the language of his correspondents. In the present paper, for example, we find, in one paragraph, the following clumsy and inaccurate phrases, among others of a like kind: "The patient *was placed in a large linseed poultice,* and, on seeing him again the following day, I was delighted to find the spongy, *irregular bottom succeeded by a more firm structure.* . . . Both *cul de sacs* merged into one. . . . Four more applications, *spread over a fortnight,* had the effect of *bringing down the sinus to the surface, &c.*"]

*Dublin Journal. May,* 1837.

---

*Medical Problems.* By Wm. Griffin, m.d., Limerick.

[This ingenious and well-written essay is occasioned by a review of Dr. Marshall's work on Spinal Irritation, in the second Number of this Journal, and is devoted to the consideration of the following questions:—"In those disorders which have gone under the name of spinal irritation, is there really any affection of the spinal cord or its membranes? or in what tissue or organ does the complaint, such as it may be, absolutely reside?" Our space will not allow us to enter, at any length, into the consideration of this subject at present; but we hope to have another opportunity of convincing Dr. Griffin that his views and our own only differ in degree, not in kind. It is true, as Dr. G. here remarks, that our "strongest objections applied more to the irrelevance or inaptness of many of Dr. Marshall's cases than to the principle he advocates;" and we would add, that our objections to Dr. Griffin's views rest a good deal on our belief, founded on considerable observation and experience, that more stress is laid, by Dr. G., on the local tenderness of the spine, as indicating the precise seat of the disease, than an enlarged and rigid induction warrants. That the great majority of the most striking symptoms of the cases referred by Dr. G. to spinal irritation, (using this term as indicating some unknown state of some part or other of the nervous centres generally, and of the spinal marrow in particular,) are justly so referred by him, we are not disposed to question; but we wish to see *the whole disease* considered philosophically, and the *spinal irritation* reduced to its proper value as a part, and not be regarded as all that is to require the attention of the practitioner; as if in this consisted the primary source and essence of the affection. We are aware that Dr. Griffin himself does not take this exclusive view of the cases, but many of his followers do; and, certainly, the great prominence which he has given in his writings to the term "spinal irritation," and in his practice to "tenderness of the spine," as indicating the precise seat of the irritation, have been productive, in our opinion, not merely of too limited pathological views, but of much ineffective and even injurious practice. We should be most thankful to receive from the pen of Dr. Griffin a work devoted to the consideration of the whole class of cases in question, viewed in their wider and, as we believe, truer bearing, as affections of the *Nervous System;* tracing them to their manifold sources in moral and physical influences, directly or sympathetically affecting the great nervous centres generally, and the spinal marrow more particularly; and we feel assured that, if he will do so, he will find that his views and ours do not very essentially differ. The subject is one of extreme interest, physiologically, pathologically, and practically, and involves the consideration of some of the most difficult problems of medical science. To the intelligent physician who witnesses the great mistakes daily committed in the diagnosis and management of such diseases, the pain and misery of which they are the source when best understood,—the singular phenomena presented by them in those protracted cases where we see young women confined to their bed or couch by them for years,—*many years,*—and yet arise almost suddenly, as if cured by enchantment,—there can be no more inviting subject; and we think that the industry and talents already evinced by Dr. Griffin in a more partial study of the subject, justify the belief that he is qualified to deal with it in the enlarged and more philosophical sense in which we think it should be regarded.

That we may not appear to withhold all the strong evidence adduced by Dr. G. in corroboration of his own views, we extract the following table and brief remarks, from the paper now before us:]

"*Summary of Cases of Spinal Irritations.*

| | CASES. | PROMINENT SYMPTOMS. |
|---|---|---|
| A. | 28 cases of cervical tenderness: 8 men. 8 married women. 12 unmarried. | Headach, nausea or vomiting, face-ach, fits of insensibility, cough, dyspnœa, affections of the upper extremities. In two cases only, pain of stomach. In five, nausea or vomiting. |
| B. | 46 cases of cervical and dorsal tenderness: 7 men. 15 married women. 24 unmarried. | In addition to the foregoing symptoms, pain of stomach and sides, pyrosis, palpitation. In thirty-four cases, pain of stomach. In ten, nausea or vomiting. |
| C. | 23 cases of dorsal tenderness: 4 men. 6 married women. 13 unmarried. | Pain in the stomach or side, cough, oppression, fits of syncope, hiccup, eructations. In one case only, nausea or vomiting. In almost all, pain of stomach. |
| D. | 15 cases of dorsal and lumbar: 1 man. 11 married women. 3 unmarried. | Pains in the abdomen, loins, hips, lower extremities, dysury, ischury, in addition to the symptoms attendant on dorsal tenderness. In one case only, nausea. |
| E. | 13 cases of lumbar tenderness. | Pains in the lower part of the abdomen, dysury, ischury; pains in the testes or lower extremities, or disposition to paralysis. In one case only, spasms of stomach and retching. |
| F. | 23 cases, all the spine tender: 4 men. 4 married women. 15 unmarried. | The symptoms of all the foregoing cases combined. |
| G. | 5 cases, no tenderness of spine. | Symptoms resembling the foregoing. |

"In all making 148 cases; twenty-six of which were males, forty-nine married women, and seventy-three girls.

"Now, why is it that none of those twenty-eight who had tenderness of the cervical vertebræ, or of the forty-six who had tenderness of the cervical and dorsal, or of the twenty-three who had tenderness of the dorsal only, by chance or design, never complained of pain in the lower part of the abdomen, loins, hips, pubis, or lower extremities, or of ischury, or dysury, or hysteralgia? And, on the other hand, why is it that none of the thirteen patients affected with lumbar tenderness complained of nausea or pain of stomach, or cough, or oppression, or affections of the upper extremities." . . . "This tenderness is found to be very generally an accompanying symptom of the disease, but by no means a necessary one. It is wanting in many cases in which the result proved organic disease of the cord was going on, and it is sometimes absent in cases of mere irritation, not only in those in which the internal affection has continued after the tenderness has been removed by remedies, but in which it never at any time existed. Thus we often find in cases manifestly of this nature, where there is yet no soreness of spine, that pain of side, or chest, or abdomen, or cough, or oppression, is produced by pressure on a particular vertebra. Tenderness of spine is a symptom of value as regards some points of diagnosis; but the doctrine and chief points of the existence of a state of disorder in the spinal cord, called irritation, preceded its discovery, and are independent of its presence."

*Dublin Journal. March,* 1837.

---

*Diabetes cured by Diuretics.* By HENRY SNOWDEN, Esq., Hull.

MR. S. was led to employ diuretics in this case from considering that, in diabetes, there is always found an excessive development of the capillaries of the kidneys, which development he believes to be of the atonic kind, favouring secretion through passive congestion of the blood in them, and thus giving rise to the increased flow of urine: diuretics, he hoped, would act beneficially by stimulating the weak and distended vessels of the kidney.—A lad, æt. 17, entered the Hull Infirmary, on the 6th October, 1836, affected with diabetes (insipidus) of ten months' standing; the quantity of urine, on admission, being sixteen pints and the drink ten pints. Venesection, diaphoretics, tonics, &c., with animal diet, were tried in vain. On the 10th of February, 1837, the quantity of water being then twenty pints, he began to take the following medicines:—℞. Pot. Nit. ℈iv.; Aquæ, ℥viij. M. cap. coch. ij. ampla ter in die, with a drachm of Sp. Æth. Nit. at bedtime, and a solution of Bitartrate of Potass for ordinary drink. This treatment was continued until the 2d March, after which he took only a little nitric ether in infusion of quassia. On the 16th March he was discharged cured, the urine having progressively decreased from the third day after beginning the diuretics until the day on which he left the hospital, when it was only three and a half pints.

[We would not advise our younger readers to reckon too much on this fortunate illustration of the *similia similibus* principle; but it merits commemoration.]

*Med. Gazette. April* 22, 1837.

---

*Case of severe Cough, ending in Rupture of the Lung and general Emphysema.* By F. G. HICKS, Esq.

A CHILD, aged ten months, after suffering about a fortnight with severe spasmodic cough, (probably hooping cough,) shewed signs of emphysema over the thorax, neck, and abdomen, and died shortly afterwards. On examination after death, the lungs were found to have given way at the root of the upper lobe of the right lung. *Ibid.*

---

*On the Use of the Nitro-muriatic Bath.* By C. LENDRICK, M.D. T.C.D. &c. Dublin.

THE object of the present paper is to recommend, as a *general* bath, the partial bath many years since introduced into practice by Dr. Scott. Dr. L. was induced to employ this bath from witnessing, many years ago, extremely good effects from it when accidentally recommended by him; and in this essay he communicates his experience of its utility in several diseases. It is administered exactly as an ordinary warm bath, at a temperature of from 90° to 95°, twice or thrice a week, and for a period of fifteen or twenty minutes. To each bath (of from thirty to forty gallons of water,) from an ounce and a half to two ounces of strong nitric acid, and from two to three of muriatic acid, are added. The practice may be persevered in for months, and is strongly recommended by Dr. L. in that large class of cachetic diseases in which mercury seems indicated, but on which it often acts as a poison instead of a remedy, in the syphiloid cachexia, the mercurial cachexia, &c., and especially in the affection "known to the public by the name of *liver* consumption." This disease, Dr. L. says, occurs generally in scrofulous habits, and may be connected with tuberculous deposit in the liver. There is a short, dry cough, loss of flesh, pain in the region of the liver, &c. It is one of the forms of the affection termed dyspeptic phthisis by Dr. W. Philip. In such cases mercury is often used, and with the worst effects; while the nitro-muriatic bath is, according to Dr. L.'s experience, most salutary. "The hepatic, and even the pulmonary symptoms, seem to yield to its influence, and the patient regains flesh and strength."

[From our own experience of the simple warm bath, and the nitro-muriatic *foot-bath*, in such cases, we are disposed to think most favorably of the more extensive application of the latter remedy recommended by Dr. Lendrick, and consider the profession indebted to him for calling the attention of practitioners to it. However, we believe Dr. L. will find, on enquiry, that the general acid bath is more used in this country than he seems to be aware.]

*Dublin Journal. May*, 1837.

---

*The History of a very extraordinary and unusually violent Case, (supposed to have been engendered by Glanders,) which terminated fatally.* By A. BROWN, Surgeon, 2d Dragoon Guards.

As the title implies, the exact nature and origin of this case are not certain. A man, who had charge of a glandered horse, and who, on the animal being killed, skinned him and exerted himself a good deal in cutting up and burying the carcass, was attacked, the same night, with rigors, headach, irritability of stomach, followed by severe continued pains and stiffness in all the large joints, aggravated on the least motion. These pains continued in an extreme degree, and were followed by the appearance, on various parts of the body, of circumscribed tumours, which were hard, livid, and insensible to the touch. These tumours came on in succession, in different parts of the body: they exhibited at first a puffy swelling, which after the lapse of twelve or fifteen hours, became first of a vermilion colour, and then dark-brown; the integuments over them became thick and callous, with superficial fissures discharging a thin acrid sanies. Before death, several of them seemed running into gangrene. About the eleventh day of the disease, a crop of warty-looking pustules appeared on different parts of the body. During the course of the disease, the right nostril was observed to be filled with a thick discharge, and the fauces were inflamed and of a purple colour. The pains continued excruciating and incessant until death, which took place on the thirteenth day of the disease.

On examination the warty-looking pustules were found to be merely elevations of the cuticle containing a thick, violet-coloured lymph; and the livid tumours all presented the following singular state of parts: the soft parts, including the muscles down to the bone, were decomposed, of a dark colour, fetid, and interspersed with small purulent deposits; the subjacent bone was "covered by a cluster of grey, circular tubercles, *the whole composed of fine cellular tissue enveloped in small cysts*,* and firmly attached to the periosteum." The Schneiderian membrane appeared throughout pale and thickened, and in one of the frontal sinuses there was a cluster of what a veterinary surgeon, who attended the dissection, considered to be "well-defined, ulcerated tubercles," and exactly similar in appearance to those found in the same cavities "in *acute* glanders in the horse."

[There seems every reason to believe that this disease derived its origin from the glandered horse, either during its lifetime or from its carcass. Mr. Brown is mistaken in believing that his case is peculiar in the disease being communicated otherwise than by the contact of morbid matter with the surface of incised or lacerated wounds." In our third Number, p. 241, he will find an account of several cases, not very dissimilar, in some of which the poison appears to have had no other mode of conveyance than one of those supposed in the case related by him.]

*Dublin Journal. May*, 1837.

* We do not quite understand the meaning of the words which we have put in Italics, as applied to tubercles.—ED.

